# Supplementary material for: easyFulcrum: An R package to process and analyze ecological sampling data generated using the Fulcrum mobile application
Source: PLoS One. 2021 Oct 6;16(10):e0254293. doi: 10.1371/journal.pone.0254293 (PMC8494344; doi:10.1371/journal.pone.0254293)
Supplement: S1 File — (HTML) [file pone.0254293.s002.html]

Project Report


# Project Report

#### Matteo Di Bernardo

#### 10 Aug 2021

## Project overview

## Collections

The first of these tables describes the presence of nematodes on a collection plate, where “yes” signifies that worms are present, “no” signifies that no worms are present, and “tracks” denotes that only tracks are observed, but no worms were found.

Total number of distinct collections: 2245

### All collections table

### All collections map

*red points* are collections with worms on sample.   
 *blue points* are collections without worms on sample.   
 *orange points* are collections with tracks on sample.   
 *black points* are collections that were NA.

### All collections plots

## Isolations

Total number of distinct isolations: 2526

## Selfing *Caenorhabditis* species

### Selfing *Caenorhabditis* table

### Selfing *Caenorhabditis* map

### Selfing *Caenorhabditis* plots

### Selfing *Caenorhabditis* species

#### C. elegans

#### C. briggsae

#### C. tropicalis
