# Supplementary material for: easyFulcrum: An R package to process and analyze ecological sampling data generated using the Fulcrum mobile application
Source: PLoS One. 2021 Oct 6;16(10):e0254293. doi: 10.1371/journal.pone.0254293 (PMC8494344; doi:10.1371/journal.pone.0254293)
Supplement: Attachment — Submitted filename: easyFulcrum Reviewers Responses.pdf [file pone.0254293.s003.pdf]

Dear Dr. Bukhari and anonymous reviewers,

Thank you for taking the time to review our manuscript. We have addressed all of the reviewers' comments. The reviewers' comments are in **bold** and our responses are in normal face. We believe that Reviewer #2 might have reviewed a different manuscript because many of the comments did not apply to this manuscript. Regardless, we responded as best as we could.

Reviewer #1: The paper by Bernardo et al. explains the data-collection platform Fulcrum and easyFulcrum applications to organize ecological sample data collection. These applications are easy to use using mobile devices, and helps with identification of microscopic organisms based on their location, photographs and genotyping. This paper focuses on easyFulcrum R package which can analyze exported data from Fulcrum and process the data by combining geographical and sequencing data. These applications are designed for nematodes but can be used for any other organism.

Review Points:
1. In line 53 authors explain about Fulcrum but no reference paper is added.

The Fulcrum data-collection application does not have a published reference in the literature. Fulcrum is a commercial product. However, users can make agreements for educational use. We've clarified this point in the manuscript where we first introduce Fulcrum.

Line 54: "It [Fulcrum] is a commercial application but can be used under a no-cost educational agreement."

2. In line 126, authors mention Genotyping template (google sheet) to enter data for isolated samples. Author explain well how to duplicate and use the template but fail to explain columns like ssu_pcr_date, its2_pcr_date etc. Also, there is no mention of the column name to input sanger sequencing data.

We agree that the descriptions for the columns in our genotyping sheet were lacking. We have addressed this issue in multiple ways. First, we revised easyFulcrum code to help non-nematode users that wish to work with a simple, generalizable genotyping sheet (six variables rather than 38). Second, we include Table 2, which describes each of the variables in the "general" genotyping sheet and how to use them. Third, we have included similar details for the larger, nematode specific genotyping sheet (see the column descriptions tab).

We have also tried to address the concern that there is no column to input sanger sequencing data in the genotyping sheet. We include new text in the "Specimen identification" methods section emphasizing that users are to generate specimen sequence data and perform alignments to sequence databases outside of easyFulcrum then enter the results from the alignments into the genotyping sheet by inputting the species name of the top alignment. We also include more details about how we perform nematode-specific species identification.

3. Authors need to explain how to input genotyping data and blast results if any new species is identified.

Thank you for pointing out this point of confusion. We included additional text to help users input genotyping data into the genotyping sheet (see methods, Specimen identification). We also included Table 2 that describes each of the variables in the genotyping sheet and explicitly addresses how to enter data regarding potential new species.

4. In line 176, author is suggesting to see methods. It will be helpful if authors mention which subheading in the methods to look for.

Agreed. We added the subheading at line 174. Also, we now refer to specific subheadings throughout the manuscript.

Line 174: "(see methods, Specimen identification)."
Line 131: "(see methods, Fulcrum data export)."

Figure 2 caption: "(see Methods, Fulcrum data export)"

5. Authors should mention that Figure 1 is also published on the following website:

We are not sure what the reviewer's concern is in this case, but the GitHub repository for easyFulcrum is a public repository (https://github.com/AndersenLab/easyfulcrum). We use the standard MIT license.

6. Figure 3A image quality is not good. Labels in the image are not legible.

We agree. We remade figure 3A and used the PACE system to ensure the image quality meets *PLoS ONE* publication standards.

7. For Figure 3, authors can include the "genotyping template" made for C.elegans, C.briggsae and C. tropicalis.

We agree that this example is a great idea. We have included the processed data used to make Figure 3 as a supplemental file (S1 data). We have also included the rendered report in HTML format as a supplemental file (S2 report). These files are cited in the "Generating summary and output files" Results section and in the figure legend for Fig 3.

Reviewer #2: The manuscript sounds technically poor, I have following concerns should be addressed before any decision.

1. There are some typos and grammatical errors in the manuscript. It is strongly suggested that the whole work to be carefully checked by someone has expertise in technical English writing.

We strongly believe that the manuscript does not contain typos or grammatical errors. Please point out line numbers so we can correct any errors.

2. Key contribution and novelty has not been detailed in manuscript. Please include it in the introduction section.

This manuscript describes a novel software package to process ecological sampling data using Fulcrum. The Introduction describes the state of the field, available technologies, and our solutions.

3. What are the limitations of the related works?

We describe the difficulties with current technologies in the Introduction (written, lack of robust database architectures, etc.).

4. Are there any limitations of this carried out study?

This manuscript is not a study. It is a description of a software package. Our limitations are that not every organism data type will be easily entered into this package. We have responded to reviewer #1's comments to make it more generalizable and have worked to make it less nematode-specific. Many species of microfauna, well beyond nematodes, will benefit from the easyFulcrum package.

5. How to select and optimize the user-defined parameters in the proposed model?

We do not use a model or any modeling in this manuscript.

6. There are quite a few abbreviations are used in the manuscript. It is suggested to use a table to host all the frequently used abbreviations with their descriptions to improve the readability.

We do not have any abbreviations, except function names. All function names are described.

7. Some sentences are too long to follow, it is suggested that to break them down into short but meaningful ones to make the manuscript readable.

Please let us know which sentences are too long to follow.

8. Explain the evaluation metrics and justify why those evaluation metrics are used?

We have no evaluation metrics in this manuscript.

9. It seems that the authors used images of equations, please use editable equation format.

We have no equations or images of equations in this manuscript.

10. The Related Works section is also fair, yet the criteria behind the selection of the works described should be explained.

We have no Related Works section.

11. The title is pretty deceptive and does not address the problem completely.

Please let us know how the title is deceptive. We describe how easyFulcrum should be used.

12. Every time a method/formula is used for something, it needs to be justified by either (a) prior work showing the superiority of this method, or (b) by your experiments showing its advantage over prior work methods - comparison is needed, or (c) formal proof of optimality. Please consider more prior works.

We have no formulae in this manuscript. The methods are explicitly described in each section.

13. The data is not described. Proper data description should contain the number of data items, number of parameters, distribution analysis of parameters, and of the target parameter itself for classification.

We describe where our example data originate. We provide those data. Additionally, in response to reviewer #1, we added a data dictionary for a large example data set.

We have no classification of parameter values in this manuscript.

14. The related works section is very short and no benefits from it. I suggest increasing the number of studies and add a new discussion there to show the advantage.

We have no Related Works section.

15. Method description is detailed and overall convincing, yet there is a big formatting problem with the end of this section and the beginning of the following one.

Please let us know what the formatting issue is. We will work to resolve it.

16. Figures 1-4 are low quality and very unprofessional figures please improve them.

This manuscript does not have a Figure 4. Figures 1-3 pass the PACE requirements for *PLoS ONE*. The PDF rendering used by *PLoS* journals generates low-quality images in the reviewer PDF.

17. Use Anova test to record the significant difference between performance of the proposed and existing methods.

We do not have ANOVA tests in this software package.
