# Supplementary material for: easyFulcrum: An R package to process and analyze ecological sampling data generated using the Fulcrum mobile application
Source: PLoS One. 2021 Oct 6;16(10):e0254293. doi: 10.1371/journal.pone.0254293 (PMC8494344; doi:10.1371/journal.pone.0254293)
Supplement: Attachment — Submitted filename: easyFulcrum Reviewers Responses Round 2.pdf [file pone.0254293.s004.pdf]

Dear Dr. Bukhari and anonymous reviewers,

Thank you for taking the time to review our manuscript. We have addressed all of the reviewers' comments. The reviewers' comments are in **bold** and our responses are in normal face.

Reviewer #1: In this study authors are explaining how to use the R-package apps Fulcrum and easyFulcrum which have been used by them in previous study published in eLife (https://elifesciences.org/articles/50465). In this paper authors introduce easyFulcrum to combine Fulcrum data with genotyping data to make sampling easy.

1. easyFulcrum installation in the methods can be more elaborated to make it user friendly. Authors can simplify how to install R from devtools::install_github ("AndersenLab/easyfulcrum"), Imager and other prerequisites.

We simplified the installation instructions to improve clarity (see Methods, easyFulcrum installation). The only requirement external to R is specific to OS X users; they need to install XQuartz outside of R. Now, we describe this clearly and include a link directly to XQuartz software. We also eliminated the unnecessary sentence describing "development prerequisites" with a link to instructions for installing command-line tools because these tools are no longer needed. We also added support for novice R users by directing them to resources for installing R and RStudio.

2. Genotyping templates have the option of entering Blast results but authors do not explain how the actual sequences of the PCR products linked to the C- and S- label in fulcrum.

We do not intend for the users to enter actual Sanger sequence data into the genotyping template. Only the name of the taxa that best aligns with the specimen is to be entered into the genotyping sheet. We have tried to clarify this point in the "Specimen identification" section of the Methods with the following text.

"We emphasize that users should generate specimen sequence data and perform alignments to sequence databases outside of easyFulcrum. Then, these species identification labels should be entered into the genotyping sheet (*e.g.*, enter the genus and species name with the best alignment score to the specimen in the species_id column."

The actual Sanger sequence data and alignments are not linked to the S-label in the genotyping Google sheet; only the name of the genus and species with the highest alignment score is added. However, the name of the species in the species_id column is linked to the S-labels using the s_label column in the genotyping sheet. All of the genotyping data are then linked back to the Fulcrum data with the *joinGenoFulc* function, which joins the two data sets by the s_label variable present in both the Genotyping and Fulcrum data. These steps are described in the "Reading, processing, and joining genotyping" section of the results.

3. Authors should mention examples of Molecular barcodes that can be used to identify organisms like DNA or RNA sequencing and lipid profiling etc.

We agree that examples of molecular barcodes would be useful for non-nematode users, but rather than listing the many options available we have added a reference to a review on the history, construction, and use of molecular barcodes in ecology and evolution (Kress et al. 2015) so that readers can design their own barcode assays. This addition was made to the end of the Specimen identification Methods section.

Kress WJ, García-Robledo C, Uriarte M, Erickson DL. **2015**. DNA barcodes for ecology, evolution, and conservation. *Trends Ecol Evol* 30:25–35.

Reviewer #2: The manuscript is relatively improved. Moreover, the manuscript is in a better format in terms of technicality and other results. I suggest accepting it.

Thank you for your feedback!
